# Bioorthogonal Non-canonical Amino Acid Tagging Combined With Flow Cytometry for Determination of Activity in Aquatic Microorganisms

**DOI:** 10.3389/fmicb.2020.01929

**Published:** 2020-08-18

**Authors:** Mathilde Lindivat, Aud Larsen, Ole Kristian Hess-Erga, Gunnar Bratbak, Ingunn Alne Hoell

**Affiliations:** ^1^Faculty of Engineering and Science, Institute of Safety Chemistry and Biomedical Laboratory Sciences, Western Norway University of Applied Sciences, Haugesund, Norway; ^2^NORCE Environment, NORCE Norwegian Research Centre AS, Bergen, Norway; ^3^Department of Biological Sciences, University of Bergen, Bergen, Norway; ^4^Norwegian Institute for Water Research, Bergen, Norway

**Keywords:** flow cytometry, protein synthesis, bioorthogonal non-canonical amino acid tagging, marine microbes, single cell activity

## Abstract

In this study, we have combined bioorthogonal non-canonical amino acid tagging (BONCAT) and flow cytometry (FCM) analysis, and we demonstrate the applicability of the method for marine prokaryotes. Enumeration of active marine bacteria was performed by combining the DNA stain SYBR Green with detection of protein production with BONCAT. After optimization of incubation condition and substrate concentration on monoculture of *Escherichia coli*, we applied and modified the method to natural marine samples. We found that between 10 and 30% of prokaryotes in natural communities were active. The method is replicable, fast, and allow high sample throughput when using FCM. We conclude that the combination of BONCAT and FCM is an alternative to current methods for quantifying active populations in aquatic environments.

## Introduction

The standard approach for assessing heterotrophic prokaryotic production in natural ecosystems is to measure incorporation of radioactive labeled leucine or thymidine (Bell, 1993; Kirchman, 1993). These methods report activity in terms of substrate uptake and/or incorporation or number of new cells produced per unit of time and volume, respectively. They have indeed been instrumental for establishing the role of bacteria in natural food webs. As bulk measurements, the values relate to the prokaryotic community as a whole. Bacterioplankton communities in natural aquatic ecosystems are, however, generally diverse and made up of many different populations. Each of them grows at their own pace depending on their physiological abilities and how well they are adapted and able to cope with the environment in which they live (Alonso-Sáez and Gasol, 2007; Del Giorgio and Gasol, 2008). Knowing how growth and activity varies and how they are distributed allow for a better understanding of the heterogeneity of microbial communities and how they function in the ecosystem.

A number of different methods have been used to investigate the vitality or fraction of active cells in prokaryote communities. These include microautoradiography to visualize incorporation of radioactive labeled substrates (Cottrell and Kirchman, 2004; Sintes and Herndl, 2006), different fluorescent stains that assesses cell membrane integrity or intracellular enzyme activity (Manini and Danovaro, 2006; Del Giorgio and Gasol, 2008), and use of tetrazolium redox dyes like CTC (5-cyano-2,3,-ditolyl tetrazolium chloride) that are reduced to fluorescent formazan in respiring cells (Rodriguez et al., 1992; Smith Erik, 1998). However, some of these methods, like the CTC and Redox assays, have shown biased results when trying to measure cell activity rates (Ullrich et al., 1999; Servais et al., 2001; Hammes et al., 2011; Netuschil et al., 2014; Emerson et al., 2017; Hatzenpichler et al., 2020). More recently, incorporation of amino acid analogues has been used to show protein synthesis in natural prokaryote communities by a click chemistry protocol termed bioorthogonal noncanonical amino acid tagging (BONCAT; Hatzenpichler et al., 2014). In short, microbial assemblages are incubated with azide‐ or alkyne-bearing methionine analogs [i.e., L-azidohomoalanine (AHA) or l-homopropargylglycine (HPG)]. Analogues incorporated into newly synthetized proteins are then made fluorescent by conjugation to alkyne‐ or azide-labeled fluorophores in a copper (I) catalyzed azide-alkyne cycloaddition chemistry reaction (a click reaction). Cells with active protein synthesis are hence made visible by standard epifluorescence microscopy (Hatzenpichler et al., 2014; Hatzenpichler and Orphan, 2015).

Bioorthogonal noncanonical amino acid tagging has successfully been used to measure activity in approximately 30 members of cultured and uncultured prokaryote phyla (Hatzenpichler et al., 2020). This suggests that BONCAT can be broadly applied to taxonomically different microorganisms. The applicability of the method has also been tested on prokaryote communities in freshwater and freshwater sediments, as well as in different marine systems and deep sea sediments (Hatzenpichler et al., 2014, 2016; Samo et al., 2014; Hatzenpichler and Orphan, 2015; Leizeaga et al., 2017). BONCAT has, in addition, been used to follow protein synthesis during viral infections of both bacteria and marine phytoplankton (i.e., *Escherichia coli*/T7, *Synechococcus* sp. WH8101/Syn1, and *Emiliania huxleyi*/EhV207; Pasulka et al., 2018) and to study microbial activity in soil communities (Couradeau et al., 2019) or the response of bathypelagic prokaryotes to starvation (Sebastián et al., 2019).

**Figure 1:**
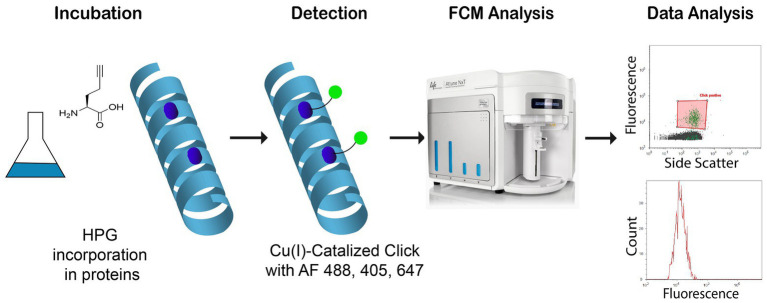
Study of newly synthetized proteins with l-homopropargylglycine (HPG) incorporation into new bacterial proteins. After labeling by click chemistry and Alexa Fluor®, azide 488, 405, and 647, samples are analyzed with flow cytometry (Blue, Violet, and Red lasers).

Flow cytometry (FCM) has become a widely used method for quantifying and analyzing aquatic microorganism, including phytoplankton, heterotrophic flagellates, bacteria, and viruses (Marie et al., 1997, 1999; Manti et al., 2012). In comparison to microscopy, FCM is more reliable and reproducible because there is less bias by operator experience and it is faster with lower running cost allowing for a much higher sample throughput. In addition, it may allow for fluorescence-activated cell sorting (FACS) and further interrogation of specific subpopulations of interest. Combining FCM and BONCAT should thus be advantageous and provide a robust method for microbial activity analysis with FCM. The usefulness has indeed been demonstrated by sorting of BONCAT labeled cells (BONCAT-FACS) for identification of active cells in complex samples (Hatzenpichler et al., 2016) and soil microbial communities (Couradeau et al., 2019), but not for measuring activity in marine samples. The possibility to use different dyes for BONCAT detection, as presented in Figure 1, allows the application to different FCM setups and for different purposes such as activity monitoring.

The main aim of this work was to establish a BONCAT protocol for FCM analysis of prokaryotic activity to be used alongside a standard FCM protocol for enumeration of bacteria. Secondly the application of BONCAT-FCM to marine microorganisms was evaluated. The protocol was developed and optimized using pure bacterial cultures and mixed marine bacterial communities, and we used different combinations of stains for total count and protein production to allow analysis for instruments with different laser combinations.

## Materials and Methods

### Incubation of Model Species and Natural Marine Bacterioplankton

**Figure 2:**
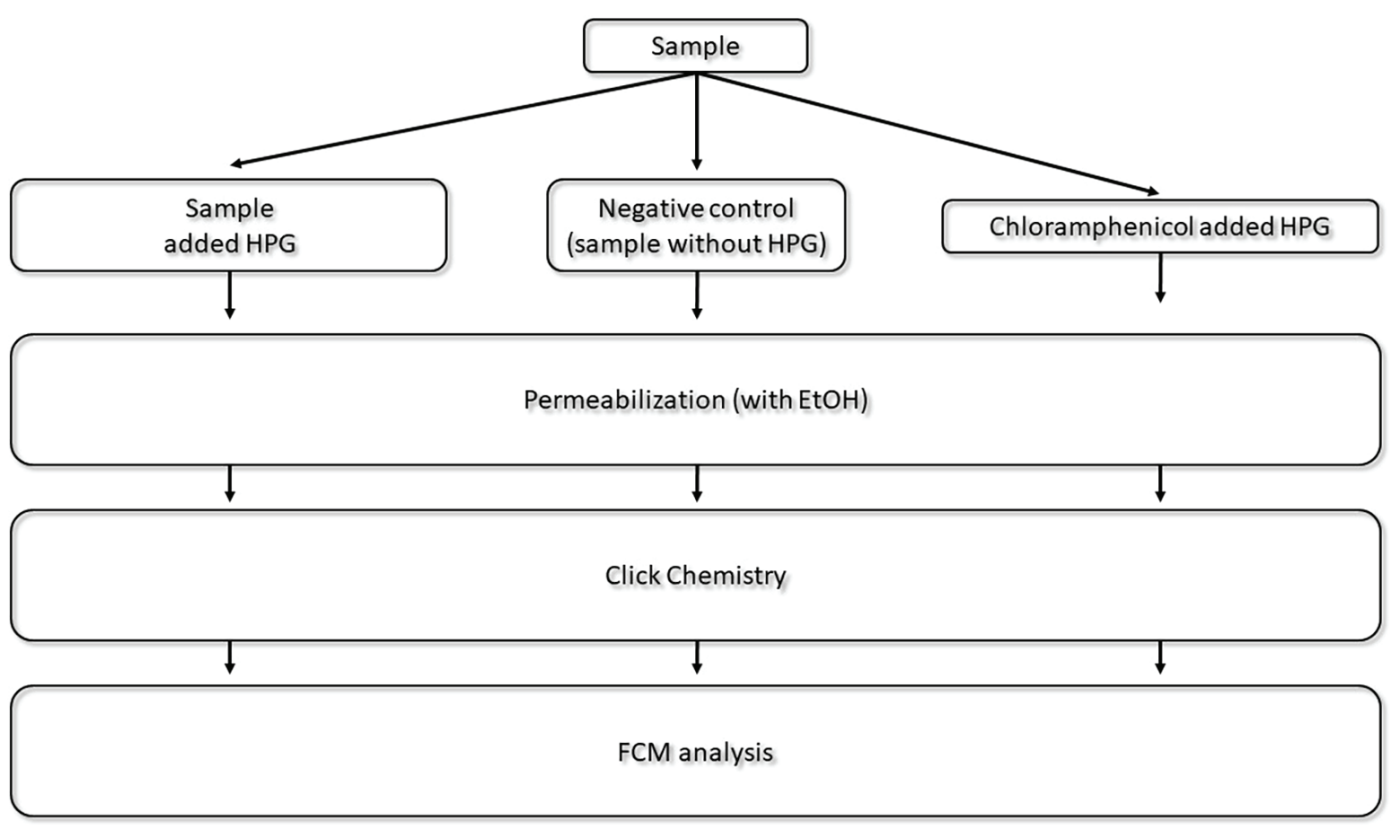
Experimental set up for natural sea water analysis with bioorthogonal non-canonical amino acid tagging. l-homopropargylglycine was used as analogous amino acid. Samples were incubated with HPG, without HPG (negative control), and with HPG and antibiotics (Chloramphenicol control) under the same conditions. After fixation with formaldehyde to stop the HPG incorporation process, samples were permeabilized. The click chemistry reaction labeled active cells with protein synthesis and detection was carried out with flow cytometry (FCM).

*Escherichia coli* (ATCC 25922) was used as model strains in this study. *E. coli* was cultured in M9 minimal media [0.5 g NaCl, 2 g glucose, 1 g NH_4_Cl, 12.8 g Na_2_HPO_4_∙7H_2_O, 3 g KH_2_PO_4_, 0.492 g MgSO_4_∙7H_2_O, 0.11 g CaCl_2_, and 0.1 g Thiamine pr. 1 L milliQ water (all chemicals purchased from Merck, Germany)], and incubated at 37°C. Natural marine microbial communities from surface water (<5 m) were collected in Karmsundet (Haugesund, Norway) and Puddefjorden (Bergen, Norway) between June 2017 and August 2019. Sea water was prefiltered at 100 μm to avoid instrument clogging. Incubations were set up directly after sampling as show in experimental set up in Figure 2.

Stock solutions of 100 mM l-homopropargylglycine (HPG) pH 7.0 (Click Chemistry Tools, USA) were prepared in pure DMSO, sterile 0.2 μm-filtered and kept at 4°C. HPG diluted in milliQ water was added to pure cultures when they reached exponential growth phase (~0.3–0.4 OD) at a final concentration of 20 nM, 50 nM, 1 μM, and 5 μM in triplicate samples. Negative control samples were prepared without HPG. Twenty five micromolar of chloramphenicol (Sigma Aldrich, USA) was added to stop protein production in control samples with HPG (Chloramphenicol control). *E. coli* and marine samples were subsequently incubated in the dark from 1–24 h and 1–48 h, respectively. After incubation, samples were fixed with 0.2 μm-filtered formaldehyde (Sigma Aldrich, USA) to a final concentration of 3% before storing at −20°C prior to the click chemistry reaction if not analyzed directly.

### Click Chemistry Protocol Validation

A protocol adapted from the Hatzenpichler group and the Samo group (Samo et al., 2014; Hatzenpichler and Orphan, 2015) was set up with Alexa Fluor® azides 488, 405, and 647 (Thermofisher Scientific, USA). After incubation with HPG and fixation, samples were centrifuged 5 min at 16,000 × *g* (Thermo Scientific, Multifuge 3SR+). Cells were resuspended in 1 ml 0.2 μm-filtered 1x phosphate-buffered saline (PBS), pelleted by centrifugation, and then resuspended and treated using 1 ml 50% ethanol followed by 3 min incubation at room temperature (RT). Ethanol was removed after centrifugation 5 min at 16,000 × *g*. Next, incubations with ethanol 80 and 96% were carried out identically. Finally, cells were washed and suspended in 1 ml 1x PBS.

The following solutions were prepared before starting the click chemistry reaction: (1) copper sulfate (CuSO_4_∙5 H_2_O; Jena Bioscience, Germany) was diluted in MilliQ water to a final concentration of 20 mM and stored at 4°C, (2) stock solutions of 50 mM Tris [(1-hydroxypropyl-1*H*-1,2,3-triazol-4-yl)methyl]amine (THPTA; Click Chemistry Tools, USA) was prepared in MilliQ water and stored in aliquots at −20°C, and (3) fresh solution of 100 mM sodium ascorbate (Sigma Aldrich, USA) in 1x PBS and 100 mM aminoguanidine hydrochloride (Sigma Aldrich, USA) in 1x PBS.

Click chemistry reaction was performed in the dark starting with a 3 min dark incubation of the pre-mix solution containing ~100 μM CuSO_4_, 500 μM THPTA (ratio of 5:1 of CuSO_4_), and 5 μM azide dye [Alexa Fluor® 488 (AF488), Alexa Fluor® 405 (AF405) or Alexa Fluor® 647 (AF647)]. Sodium ascorbate (5 mM final concentration) and aminoguanidine hydrochloride (5 mM final concentration) were added directly to each sample, followed by 17.5 μl of pre-mix. All reactions were carried out in eppendorf tubes, in a final volume of 1.15 ml. Samples were gently mixed by turning the tube, before incubation for 30 min in the dark. After incubation, samples were centrifuged at 16,000 × *g* for 5 min, washed two times with 1x PBS, and finally suspended in sterile 1x PBS or 1x TE-buffer.

### Double Staining Procedure

Total bacterial numbers were determined by flow cytometry after DNA staining using either LDS 751 or SYBR Green. Samples with azide AF488 labeled proteins were stained with 10 μl of 20 μg/ml LDS 751 stock solution (Invitrogen, USA), followed by incubation for 10–15 min in the dark at RT. Samples with AF405 azide and AF647 azide labeled proteins were stained with 10 μl of 100x SYBR Green (Invitrogen, USA) for 10–15 min in the dark at RT, following an adaptation of Marie et al. (1999) protocol for marine samples. No washing steps were performed after the double staining procedure.

### Flow Cytometry Analysis

Flow cytometry analysis was performed with an Attune NxT Acoustic Focusing Cytometer (Thermofischer scientific, USA) with a Violet laser 405 nm (50 mW), a Blue laser 488 nm (50 mW), and a Red laser 638 nm (100 mW). Attune Performance tracking beads (2.4 and 3.2 μm; Thermofischer, USA) were used for instrument calibration. The following detectors were used for fluorescence detection according to dyes excitation and emission wavelength (Table 1): BL1 (530/30) for SYBR Green and Alexa Fluor® 488 azide (AF488), VL1 (440/50) for Alexa Fluor® 405 azide (AF405), RL1 (670/14) for Alexa Fluor® 647 azide (AF647), and LDS 751. Trigger was set at 2000 on Side Scatter SSC-H (for LDS 751) or on BL1 (for SYBR Green). Voltages were optimized for each detector. SYBR Green was used in combination with AF405 azide or AF647 azide, whereas LDS 751 was used with AF488 azide. For seawater analysis, size-standardized beads ranging from 0.5 to 2 μm (Thermofisher Scientific, USA) were used to determine bacterial and virus like particles populations. Between 200 and 2,000 cells were analyzed at a flow rate of 25 μl·min^−1^. Counts were performed on three biological replicates and each of samples were analyzed three times. Counts were also performed on higher cell numbers (20,000 and 200,000) and showed similar results than lower cell numbers analysis (200, 2,000).

**Table 1:** Overview of excitation and emission wavelength for DNA and click dyes used in the study.

Dyes	Excitation (nm)	Emission (nm)
SYBR Green	497	520
LDS 751	543	712
AF405 azide	401	421
AF488 azide	490	525
AF647 azide	650	665

### Microscopy Analysis

After the click chemistry reaction, samples were filtered onto black polycarbonate 0.2 μm filters (Whatman, UK), stained with SYBR Green, and rinsed with sterile 1x PBS. Samples were counted in a Nikon Optiphot-2 microscope (Nikon Instruments, Japan) with Hg lamp C-SHGI (Nikon Instruments, Japan). Images were obtained with NIS-Elements 2.20 (Nikon Instruments). The fluorescence filters used were B-2A (Ex450-490) for AF488 azide and SYBR Green, and G2-A (Ex510-560) for LDS 751.

## Results and Discussion

### Optimization and Application of BONCAT to FCM

**Figure 3:**
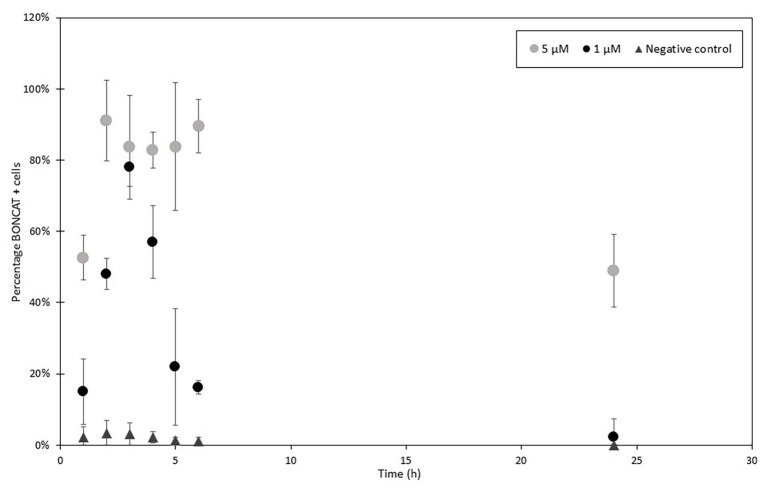
HPG incorporation in *Escherichia coli* over time with 1 μM (black) and 5 μM (gray) and control (dark gray). HPG samples were collected every hour and then at 24 h after the incubation start and analyzed with FCM and LDS 751/AF488 azide. Percentages of positive cells were determined comparing to total cell enumeration (2,000 cells analyzed per samples in triplicates). Activity increased in the first 3–4 h to decrease in the following hours. After, new produced cells do not incorporate HPG combined with a continuous increase in cell concentration leading to a diminution of detected activity.

**Figure 4:**
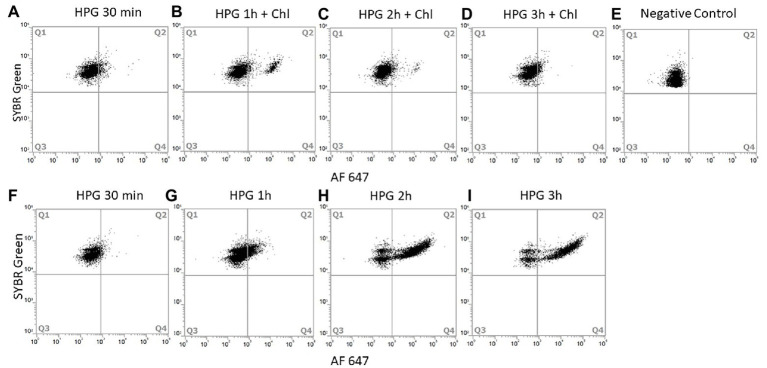
Chloramphenicol action on protein production of *E. coli*. Chloramphenicol was added after 30 min of incubation with HPG and samples were collected every hour. Cells were analyzed with SYBR Green/AF647 azide and FCM (10,000 cells analyzed). Dot plot of *E. coli* cells incubated for: 30 min with HPG **(A)**, 1 h with HPG and 30 min with chloramphenicol **(B)**, 2 h with HPG and 1.5 h with chloramphenicol **(C)**, 3 h with HPG and 2.5 h with chloramphenicol **(D)** Negative control without HPG **(E)**. Respective positive control incubation of *E. coli* with HPG for 30 min **(F)**, 1 h **(G)**, 2 h **(H)**, and 3 h **(I)**. Q1 contain negative BONCAT cells; Q2 contain positive BONCAT cells (active). Positive BONCAT cells (=active cells) increased to 6 ± 0.7% during the first 30 min of incubation with HGP **(A)**. After adding chloramphenicol, activity stabilized to 8.8 ± 1.7% **(B)** and started to decrease at 2 h **(D)** whereas the positive control increased from 3 ± 0.4% **(F)** to 18.6 ± 2% **(G)**, 71.2 ± 0.3% **(H)** and 79.6 ± 0.1% **(I)**.

Incubation conditions for the proposed method to measure activity in single bacterial cells using flow cytometry were optimized with pure *E. coli* cultures grown on minimal media M9 to deplete methionine stocks (Dieck et al., 2012) and increase l-homopropargylglycine (HPG) incorporation. BONCAT incorporation was tested for a range of HPG concentrations from 20 to 5 μM with LDS 751/AF488 azide dyes. The highest level of incorporation was observed after 1–3 h incubation time using 5 μM HPG (91% of active cells; Figure 3). Low HPG concentrations (20 and 50 nM) were not suitable for long time incubations as numbers of positive cells was low and standard deviations between three replicates were high. Depletion of HPG over time due to the increase in cell population during exponential growth will lead to a reduction in the number of detected active cells. We suggest to use high HPG concentration (>15 μM) for monoculture incubation. As reported previously (Hatzenpichler and Orphan, 2015; Hatzenpichler et al., 2016; Leizeaga et al., 2017; Pasulka et al., 2018), we did not find that high concentrations (over 1 μM) of HPG affected cell growth positively nor negatively. Negative controls without HPG showed that false positive cells varied between 0 and 13% during incubation and that a higher content of false positive click cells were observed after short (1–2 h) compared to long incubation times (up to 24 h). However, this number was influenced by the use of the LDS751/AF488 azide. Whether LDS751 was suitable for separating the bacterial population from the background noise can be questioned as we observed a much lower false positive rate for SYBR Green/AF647 azide (0.01–0.1%). Chloramphenicol controls were included in the experiments to confirm HPG incorporation only in vital cells (Samo et al., 2014). HPG incorporation stopped during the first 2 h after addition of chloramphenicol and then stabilized, verifying stop in protein production (Figure 4).

The click reaction in liquid was a critical step of the protocol. The oxidation state of Cu(I) has to be maintained during the conjugation of the analogous amino acid (Presolski et al., 2011); we therefore carried out the reaction in 1,000 μl volume. Cell pelleting and supernatant removal for washing steps and ethanol treatment caused around 11 ± 2% cell loss for *E. coli* samples and need to be done with care to limit cell loss. A greater cell loss can be expected with natural communities due to their relative small sizes. Couradeau et al. (2019) proposed an alternative method where cells were filtered, clicked, and then vortexed to resuspend the cells from the filter. The amount of cells resuspended varied between samples causing biased results. None of the methods managed to capture 100% of the cells.

**Figure 5:**
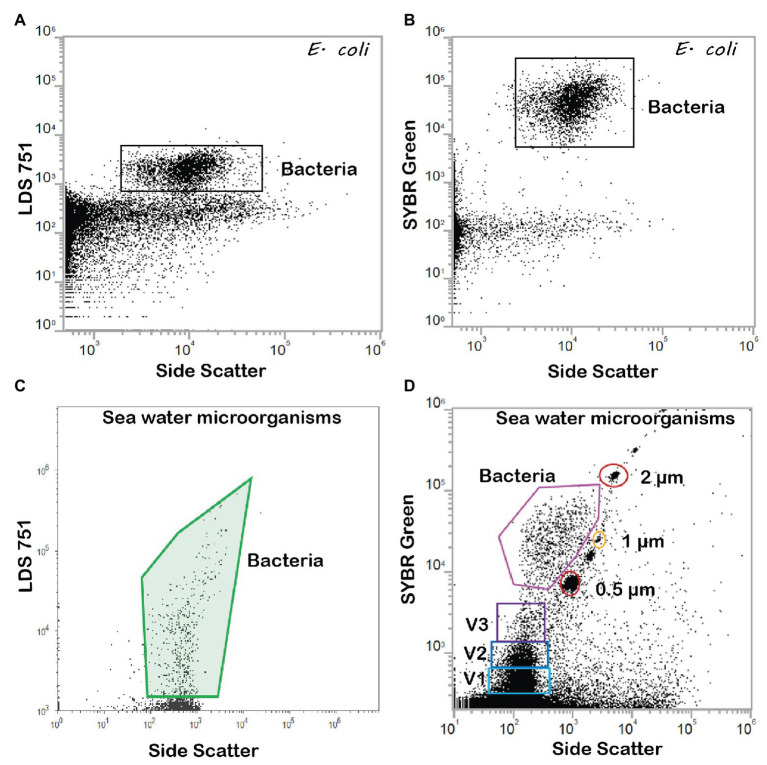
FCM dot plots of *E. coli* stained with **(A)** DNA stain LDS 751, one population and **(B)** DNA stain SYBR Green revealing two bacterial populations, high (HNA) and low DNA (LNA) content. Sea water microorganisms stained with **(C)** LDS 751 and **(D)** SYBR Green. In addition to bacteria, small particles that are consistent with the size of viruses are detected with SYBR Green (Gates V1, V2, and V3). Gates were determined for each dye and size-standardized beads of 2, 1, and 0.5 μm were used for size identification (Thermofisher Scientific, USA).

**Figure 6:**
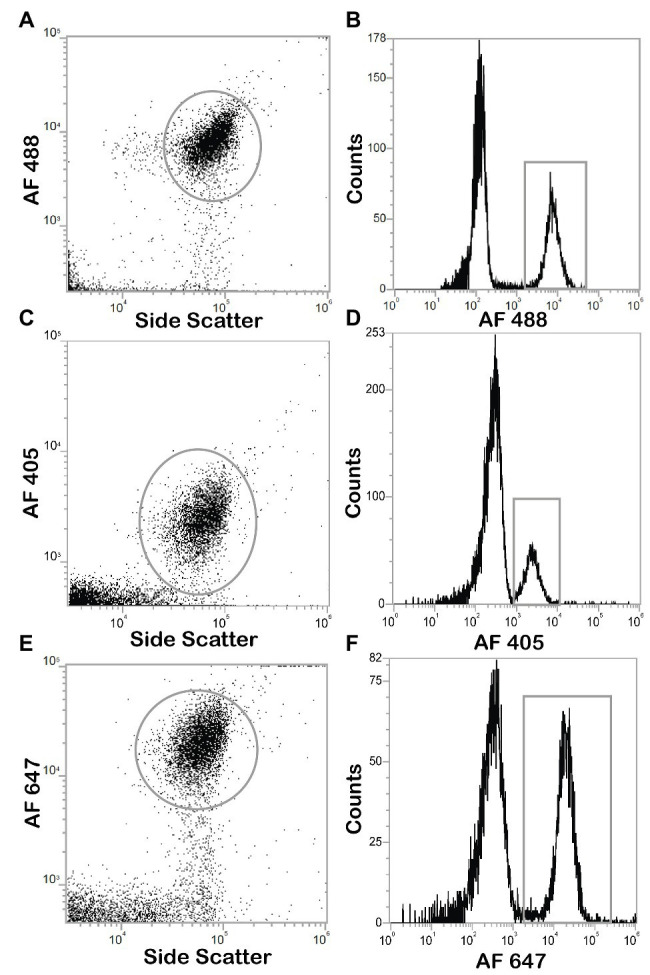
Dot plots and histograms of *E. coli* cells positives for click chemistry with Alexa Fluor® azide dyes. Cultures are incubated with HPG, permeabilized with ethanol, and detected with click chemistry, analysis with Attune NxT flow cytometer. Positive cells were determined by gating on dot plots (gray circle), the population can be found in the corresponding histogram (gray box). **(A)** Dot plot of click positive cell with AF488 azide. **(B)** Histogram of click positive cell with AF488 azide **(C)** Dot plot of click positive cell with AF405 azide. **(D)** Histogram of click positive cell with AF405 azide. **(E)** Dot plot of click positive cell with AF647 azide. **(F)** Histogram of click positive cell with AF647 azide.

**Figure 7:**
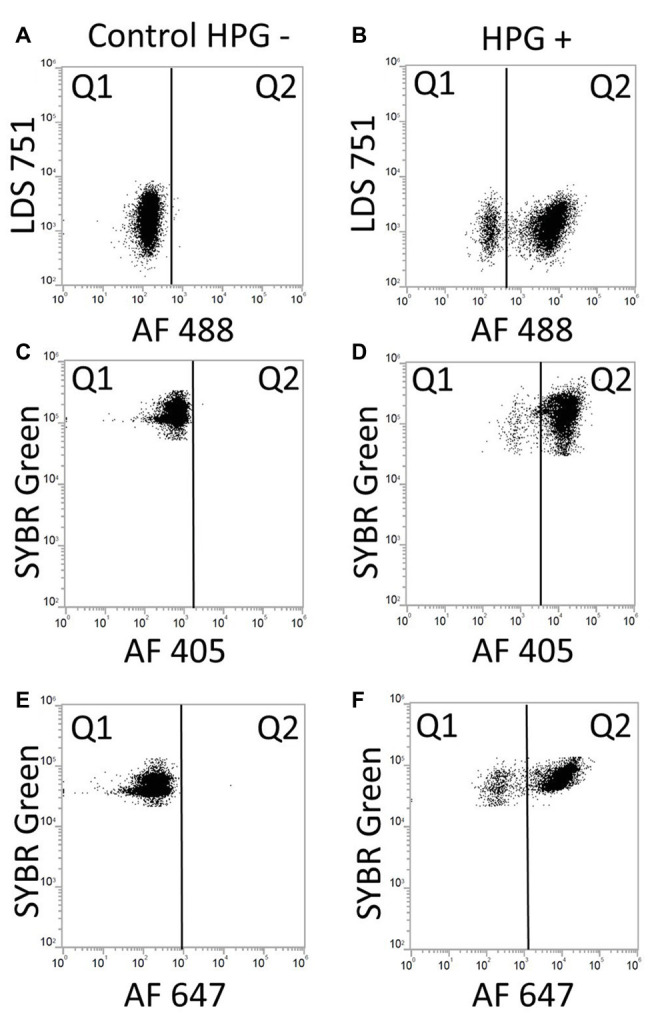
Dot plots of BONCAT/DNA staining with LDS 571/AF488 azide, SYBR Green/AF405 azide and SYBR Green/AF647 azide. Gates were positioned according to unstained samples and instrument settings. **(A)** Control negative HPG with LDS 751/AF488: LDS 751 stained cells in Q1. **(B)** Positive control HPG: LDS 751 positive cells in Q1 and Q2, positive BONCAT cells in Q2. **(C)** Control negative HPG with SYBR Green/AF405: SYBR Green stained cells in Q1. **(D)** Positive control HPG with SYBR Green/AF405: SYBR Green positive cells in Q1 and Q2, positive BONCAT cells in Q2. **(E)** Control negative HPG with SYBR Green/AF647. **(F)** Positive control HPG with SYBR Green/AF647: SYBR Green positive cells in Q1 and Q2 (AF647), positive BONCAT cells in Q2.

The two DNA stains tested, LDS751 and SYBR Green, gave comparable numbers for *E. coli* cultures (similarity of 96 ± 5.1%; Figures 5A,B). For discrimination between bacteria and background noise, as well as between various microbial populations in marine environmental samples, LDS751 (Figure 5C) performed poorer than the well-established marine microbes stain SYBR Green (Noble and Fuhrman, 1998; Marie et al., 1999). As described by Marie et al. (1999), various virus like populations (V1, V2, and V3) were observed for marine samples in addition to bacteria (Figure 5D). When comparing active vs. total *E. coli* cell numbers analyzed with FCM and microscopy counts, the results agreed to a large extent (Table 2). Nevertheless, when red emitting dyes like AF647 were used, FCM was more sensitive than epifluorescent microscopy as cells with low fluorescence that were detected by FCM were difficult to see in the microscope (data not shown). Since the performance with SYBR Green was better than LDS for marine bacteria, we chose the SYBR Green counterstaining protocol for our final BONCAT procedure. LDS 751 in combination with Alexa Fluor® 488 azide (AF488) dye was used for *E. coli* only to optimize HPG concentration. The result of the combined staining is presented in Figure 6 where the number of total active bacterial numbers, as determined by FCM, derives from the dot plots (Figures 6A,C,E) and the corresponding histograms provide the distribution of active bacterial population (Figures 6B,D,F). These plots show that AF488 azide and AF647 azide gave cells with highest fluorescence intensities that also separated well from background noise (Figures 6A,E). A difference of laser power (100 mW for red laser and 50 mW for violet laser) could explain the variation of separation observed for AF647 azide and AF405 azide. We agree with Hatzenpichler and Orphan (2015) that the optimum concentration for azide dyes in click reactions is 5 μM. Figure 7 shows the BONCAT/DNA staining for all used dyes and how quadrants gating allows to separate active from non-active cells and the background whereas Figures 6B,D,F, show how the incorporation of HPG follow a normal distribution, as expected for cells in exponential growth phase, indicating that the cells are highly active and incorporate similar amount of HPG.

**Table 2:** Comparison microscopy-flow cytometry (FCM).

Incubation time (h)	Microscopy		FCM	
	Total cells/ml	% Positive	Total cells/ml	% Positive
1	1.20E+07	34 ± 4	1.71E+07	42 ± 1
2	1.07E+07	83 ± 11	8.74E+06	96 ± 1
3	1.40E+07	63 ± 4	1.62E+07	74 ± 8
4	2.00E+07	34 ± 26	1.70E+07	61 ± 3

Epifluorescence microscopy and FCM analysis of *Escherichia coli* incorporation of l-homopropargylglycine labeled with AF488 azide and DNA stained with LDS 751.

### BONCAT, Flow Cytometry, and Activity of Marine Microbial Communities

**Figure 8:**
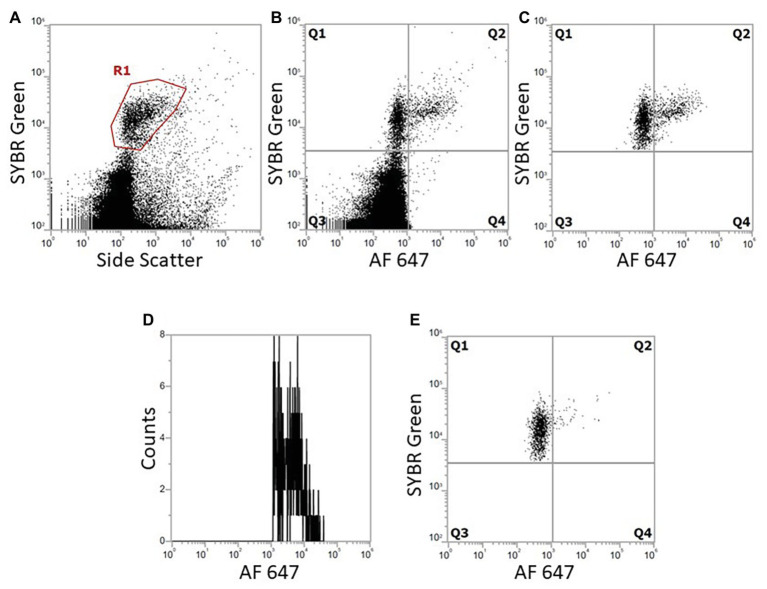
Dot plots and histogram from FCM show marine bacterial metabolic activity with BONCAT using double staining with SYBR Green and AF647 azide. Bacterial population was gated from SYBR Green/SSC. When analyzing both click and SYBR Green, all events outside the SYBR Green/SSC gate are excluded from click analysis. **(A)** DNA stained cells gated from SYBR Green fluorescence (R1). **(B)** Combination of DNA stain and BONCAT, Q1 are DNA stained bacteria, negative BONCAT; Q2: BONCAT positive specifically selected bacteria; Q3: small fluorescent particles and background; and Q4: background and unspecific BONCAT staining. **(C)** Gated bacteria from **(A)** are further analyzed with comparison with BONCAT fluorescence to select bacteria specifically (2,324 bacteria analyzed). **(D)** Histogram of Q2 cells, the distribution of positive cells depending on their fluorescence activity. **(E)** Negative control without HPG following click reaction (1,422 bacteria analyzed).

The adjusted BONCAT methodology applied on marine environmental samples produced results which show that FCM distinguishes active cells from non-active cells and background particles (Figure 8). Incubations of marine samples with HPG were carried out over 48 h and analyzed with SYBR Green-AF647 azide. We recommend AF647 azide dye for BONCAT to avoid noise contamination from the sample (observed with AF405). Prokaryotic abundances ranged from 1.6 × 10^5^ to 1.4 × 10^6^ cells/ml, which are common concentrations in Norwegian coastal waters (Table 3; Eilers et al., 2000; Larsen et al., 2004; Brakstad and Lødeng, 2005; Reinthaler et al., 2005). More importantly, activity levels (between 6 and 39% active cells) measured using BONCAT were in accordance with levels measured using other methods (esterase activity, CTC, microautoradiography etc.) which often report that 10–60% of the cells are active (Del Giorgio and Gasol, 2008). Prokaryotes growth rates and biomass production distribution characterize communities and how much they contribute to element cycling, energy flow, and other natural processes such as viral lysis (Kirchman, 2016). BONCAT combined with FCM allows identification of the active part of a community at a single cell level. This enables the exploration of activity at a higher resolution and thus facilitate further analysis to go deeper into their role in the environment.

**Table 3:** Detection of marine bacteria activity with SYBR Green/AF647 azide after incubation with HPG.

Date	Location	Incubation time (h)	Total bacteria/ml	Number of cell analyzed	Positive active cells (%)
22.10.2018	Haugesund	6	4.20E + 05	222	6.42 ± 5.60
22.10.2018	Haugesund	6	4.36E + 05	267	10.08 ± 1.49
22.10.2018	Haugesund	6	5.20E + 05	204	8.40 ± 12.40
22.10.2018	Haugesund	6	4.08E + 05	281	13.60 ± 12.40
27.11.2018	Haugesund	6	1.29E + 06	232	39.90 ± 1.56
27.11.2018	Haugesund	6	1.43E + 06	302	30.19 ± 3.67
05.02.2017	Haugesund	2	1.46E + 05	384	30.45 ± 0.11
05.02.2018	Haugesund	5	1.57E + 05	270	30.75 ± 4.64
05.02.2019	Haugesund	24	1.84E + 05	520	23.2 ± 2.4
05.02.2019	Haugesund	8	1.69E + 05	460	26.75 ± 0.8
06.09.2019	Puddefjord	1	1.80E + 05	2,324	25.1 ± 1.55

Samples were incubated in replicates. Different incubation time are represented here. In general, 6 h incubation was selected. Total bacterial number was evaluated for each sample.

One challenge when trying to describe microbial activity by FCM is that the fluorescence level of dormant or low activity cells is so low that they are masked by background noise. Our results show how the resulting underestimation of actual bacterial activity can be avoided by double staining, which enables differentiation of particles from microbes, improves the flow cytometric signal:noise ratio, and allows the determination of percentage of active cells in a population. The combination of DNA stains and activity stains allows for a two-step detection: (1) identification of microbial cells and (2) detection of active cells, and thus extract them from background noise as demonstrated in Figure 8. Hatzenpichler et al. (2016) did not use counter staining for cells sorting. For our analysis, side scatter (SSC) was selected as the second parameter to create plots for each dye. Couradeau et al. (2019) used forward scatter (FSC) in their study. Both parameters are representative of cell characteristics with FSC for cell size and SSC for cell granularity or complexity. However, SSC is more commonly used for small particle analysis (such as microbes) as the signal is detected using a more suitable photomultiplier tube (PMT) than FCS (Shapiro, 2003). SSC is also more consistent between instruments compared to FSC where variations can be observed (Gasol and Del Giorgio, 2000).

**Figure 9:**
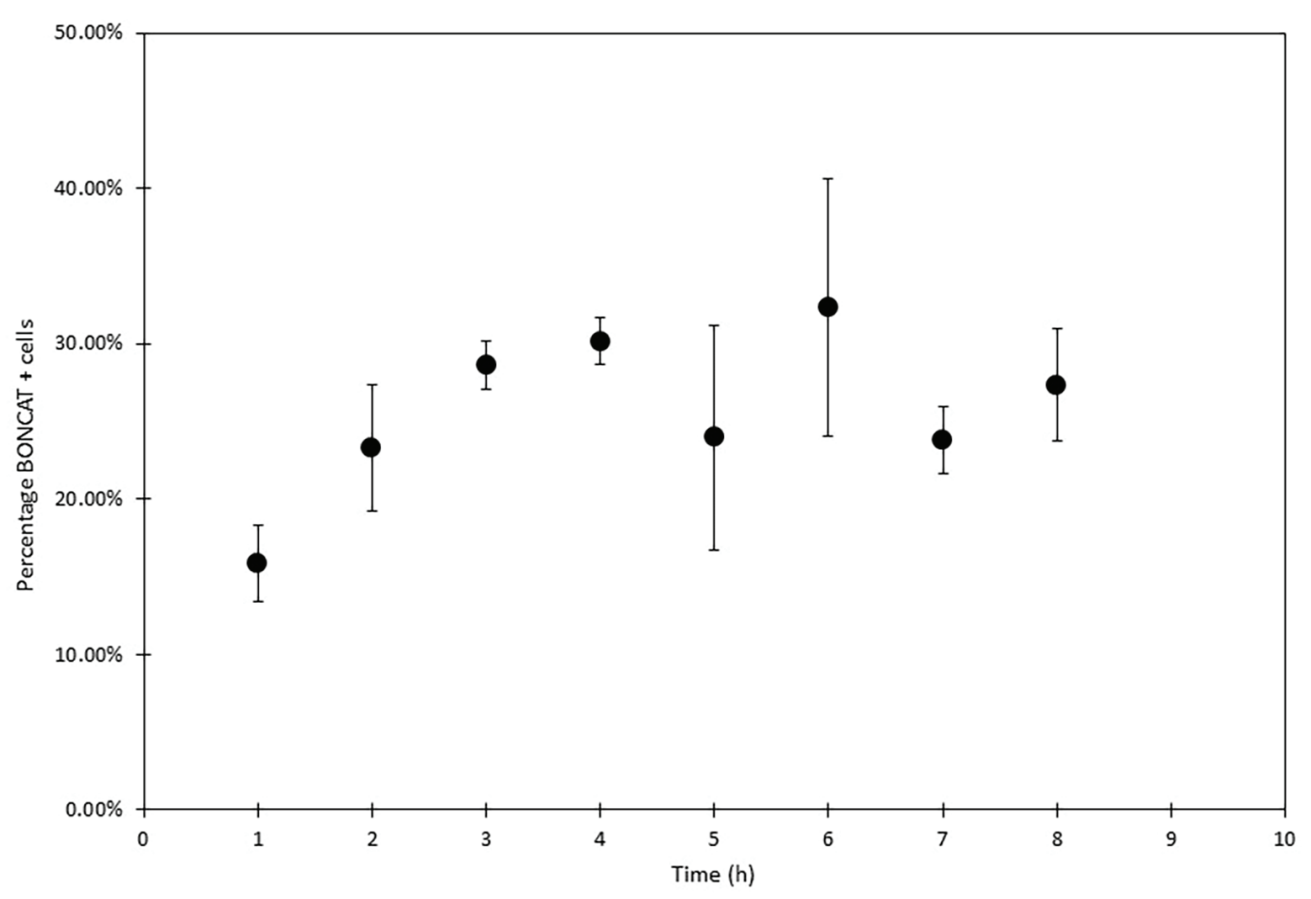
Sea water microorganisms BONCAT analysis with FCM (SYBR Green and AF647 azide) over 8 h. Protein synthesis activity was monitored every hour (in triplicates biological samples and analyzed three times). Activity increased in the first three hours to reach a stable state.

Several concentration of HPG were evaluated, and contrary to Samo et al. (2014) who used low HPG concentrations (20 nM), we found that also for marine prokaryotes, higher HPG concentrations (1 μM) increase the levels of detection for low fluorescent cells. Lower HPG concentration 20 and 50 nM produced very poor results with FCM. The optimal time for incubation with HPG can vary depending on the type of samples. For slow growing organisms from deep sea environment (Hatzenpichler and Orphan, 2015; Hatzenpichler et al., 2016), a long incubation time is necessary (>100 days). For surface water microorganisms, Leizeaga et al. (2017) proposed an incubation time of 1 or 4 h to evaluate metabolic activity in marine microbial community whereas Samo et al. (2014) selected 1 h incubation. Here, we analyzed metabolic activity over 48 h with regular sampling between 1 h and 8 h, as well as at 24 h and 48 h. Longer incubations may result in community changes that can influence the overall and initial activity. Our study showed incorporation of HPG during the first hours (1–3 h), and percentage of active cells remained stable over time (Figure 9). Several features may explain the stabilization of the fraction of labeled cells (i.e., cells above some detection threshold) including growth rate distribution, cell size (protein content) distribution, depletion of substrate, starvation or dormancy state, cellular protein turnover, and initial surplus (luxury) uptake of substrate (Del Giorgio and Gasol, 2008). Thorough studies with variation of multiple factors are necessary to interpret and understand the results obtained with BONCAT analysis when applied to natural microbial communities.

We have demonstrated the possible use of FCM for BONCAT analysis. Our results show that the combination BONCAT-DNA staining with FCM represent a reliable protocol for analysis of bacterial metabolic activity in monocultures as well as aquatic natural samples. Application of BONCAT to FCM has several advantages useful for future applications. Firstly, cell sorting of active cells, with the possibility of further analysis in environmental investigations is possible (Hatzenpichler et al., 2016; Couradeau et al., 2019). The active portion of a prokaryote community can be identified by 16S tagging (Hatzenpichler et al., 2016). A wide range of applications can be associated with cell sorting considering cells are not destroyed or damaged by the analysis. Couradeau et al. (2019) compared BONCAT+ cells identified by 16S sequencing with bacterial isolates from the same environment. Pasulka et al. (2018) reported the possibility to observe virus production with BONCAT and microscopy analysis. Virus are produced from host cells proteins, and the incorporation of HPG thus enable the detection of newly produced viral particles. The use of SYBR Green as DNA stain is appropriate for virus analysis (Marie et al., 1999; Brussaard, 2004), and the adaptation of the protocol can be used to detect FCM BONCAT positive small particles that we interpret as viruses (Figure 9). Microbial growth to understand the impact of biotic and abiotic factors may also benefit of the BONCAT-FCM method (Sebastián et al., 2019). It is also possible to follow a bacterial community over time and identify principal actors and observe community changes in an environment associated with nutrients flux. If FCM instruments are available, onboard research vessels single cell activity can be monitored. Hatzenpichler defines BONCAT as part of the next-generation physiology approach where individual cells can be analyzed specifically (Hatzenpichler et al., 2020). The possibility to focus the research on active marine prokaryotes and not the whole community gives opportunity to improve or reinterpret microbial analysis.

## Data Availability Statement

The datasets generated for this study are available on request to the corresponding author.

## Author Contributions

IAH, AL, OKH-E, and GB initiated and supervised the study. ML and GB performed the experimental part. ML wrote the manuscript, with significant inputs from GB, IAH, AL, and OKH-E. All authors contributed to the article and approved the submitted version.

### Conflict of Interest

The authors declare that the research was conducted in the absence of any commercial or financial relationships that could be construed as a potential conflict of interest.

## Abbreviations

AF: Alexa fluor
AHA: L-azidohomoalanine
BONCAT: Bioorthogonal non-canonical amino acid tagging
CTC: 5-cyano-2,3,-ditolyl tetrazolium chloride
FACS: Fluorescence-activated cell sorting
FCM: Flow cytometry method
FISH: Fluorescence *in situ* hybridization
HPG: L-homopropargylglycine
THPTA: Tris [(1-hydroxypropyl-1*H*-1,2,3-triazol-4-yl)methyl]amine
