# Supplementary material for: Bioorthogonal Non-canonical Amino Acid Tagging Combined With Flow Cytometry for Determination of Activity in Aquatic Microorganisms
Source: Front Microbiol. 2020 Aug 18;11:1929. doi: 10.3389/fmicb.2020.01929 (PMC7461810; doi:10.3389/fmicb.2020.01929)
Supplement: Supplementary file 1 [file Table_1.DOCX]

Supplementary 1

Table 1: Statistical analysis of different cell counts. 200, 2000, 20000 and 200000 cells were analysed from 3 different replicates. The percentage of positive BONCAT cells was calculated from the total of cells analysed.

| Number of cell | BONCAT + | SD |
| --- | --- | --- |
| 200 | 93% | 1% |
| 2000 | 95% | 1% |
| 20000 | 96% | 1% |
| 200000 | 98% | 0% |
